# Developmental encoding of natural sounds in the mouse auditory cortex

**DOI:** 10.1093/cercor/bhae438

**Published:** 2024-11-06

**Authors:** Stefano Zucca, Chiara La Rosa, Tommaso Fellin, Paolo Peretto, Serena Bovetti

**Affiliations:** Department of Life Sciences and Systems Biology (DBIOS), University of Turin, via Accademia Albertina 13, 10123 Turin, Italy; Neuroscience Institute Cavalieri Ottolenghi (NICO), University of Turin, Regione Gonzole 10, 10143 Orbassano, Italy; Department of Life Sciences and Systems Biology (DBIOS), University of Turin, via Accademia Albertina 13, 10123 Turin, Italy; Neuroscience Institute Cavalieri Ottolenghi (NICO), University of Turin, Regione Gonzole 10, 10143 Orbassano, Italy; Optical Approaches to Brain Function Laboratory, Istituto Italiano di Tecnologia, via Morego 30, 16163 Genoa, Italy; Department of Life Sciences and Systems Biology (DBIOS), University of Turin, via Accademia Albertina 13, 10123 Turin, Italy; Neuroscience Institute Cavalieri Ottolenghi (NICO), University of Turin, Regione Gonzole 10, 10143 Orbassano, Italy; Department of Life Sciences and Systems Biology (DBIOS), University of Turin, via Accademia Albertina 13, 10123 Turin, Italy; Neuroscience Institute Cavalieri Ottolenghi (NICO), University of Turin, Regione Gonzole 10, 10143 Orbassano, Italy

**Keywords:** auditory cortex, mouse communication, spontaneous activity, two-photon calcium imaging, ultrasonic vocalizations

## Abstract

Mice communicate through high-frequency ultrasonic vocalizations, which are crucial for social interactions such as courtship and aggression. Although ultrasonic vocalization representation has been found in adult brain areas along the auditory pathway, including the auditory cortex, no evidence is available on the neuronal representation of ultrasonic vocalizations early in life. Using in vivo two-photon calcium imaging, we analyzed auditory cortex layer 2/3 neuronal responses to USVs, pure tones (4 to 90 kHz), and high-frequency modulated sweeps from postnatal day 12 (P12) to P21. We found that ACx neurons are tuned to respond to ultrasonic vocalization syllables as early as P12 to P13, with an increasing number of responsive cells as the mouse age. By P14, while pure tone responses showed a frequency preference, no syllable preference was observed. Additionally, at P14, USVs, pure tones, and modulated sweeps activate clusters of largely nonoverlapping responsive neurons. Finally, we show that while cell correlation decreases with increasing processing of peripheral auditory stimuli, neurons responding to the same stimulus maintain highly correlated spontaneous activity after circuits have attained mature organization, forming neuronal subnetworks sharing similar functional properties.

## Introduction

Mice communicate through a variety of signals that include the emission of complex ultrasonic vocalizations (USVs), spanning a range of 35 to 110 kHz (Holy and Guo 2005). USVs display complex acoustic features, suggesting that multiple categories of vocalizations exist and differently influence distinct social behaviors (Sangiamo et al. 2020). Mouse vocalizations consist of multiple syllables, which are continuous sound units separated by brief periods of silence, organized into nonrandom patterns, often repeated in phrases (Holy and Guo 2005; Arriaga and Jarvis 2013; Agarwalla et al. 2023). Changes in the sequence of syllables, their acoustic properties, and prevalence within a phrase provide insights into specific emitter characteristics such as developmental stage, genetic strain, health, and social status (Grimsley et al. 2011; Scattoni et al. 2011; Chabout et al. 2015; Fonseca et al. 2021; Lenschow et al. 2022). Therefore, USVs play a fundamental role in many mice social behaviors such as courtship, mate selection, and aggression.

Responses to USVs have been characterized in different adult rodent brain areas, including the primary auditory cortex (ACx), as well as in higher-order ACx regions (Geissler and Ehret 2004; Carruthers et al. 2013; Rao et al. 2014; Levy et al. 2019; Calhoun et al. 2023), and both mice (Garcia et al. 2015; Afrashteh et al. 2022) and rats (Kim and Bao 2009) exhibit a tonotopic organization over representing high-frequency (HF) tones in the central auditory system, supporting the importance of encoding ethologically relevant sounds.

In altricial species, the capacity to respond to auditory stimulation develops postnatally with the opening of the auditory canal occurring around P11 in mice (Polley et al. 2013; Chen et al. 2021), although wide-field calcium imaging revealed responses before ear canal opening (Babola et al. 2018; Meng et al. 2020). Pure-tone tonotopic responses have been shown to develop rapidly between P12 and P14, reaching an adult-like representation at P14 to P16 in mice (Carrasco et al. 2013; Polley et al. 2013; Chen et al. 2021). The early development of tonotopy comes with a marked increase in dendritic complexity and spine number, which is followed by a period of spine maturation approximately after P16 (McMullen et al. 1988; Schachtele et al. 2011; Meng et al. 2020), which corresponds to the functionally identified critical period for audition (Zhang et al. 2001; Barkat et al. 2011; Meng et al. 2020).

The development of complex sound responses is slightly delayed compared with pure-tone responses and emerges in a series of sensitive periods within a month-long critical period window (Insanally et al. 2009; Insanally et al. 2010; Carrasco et al. 2013; Kim and Bao 2013). Interestingly, early exposure to complex USV frequencies is essential for the overrepresentation of HF (>45 kHz) sounds in the rat auditory cortex (Kim and Bao 2013), and, in both laboratory and wild female mice, exposure to paternal USVs during development has been shown to induce the formation of long-lasting memories guiding female mate choice in adulthood toward unfamiliar USVs (Hammerschmidt et al. 2009; Musolf et al. 2010; Asaba et al. 2014). This evidence suggests that early auditory experiences significantly impact complex sound processing at both the neuronal representational and behavioral levels, from early development through to adulthood.

Importantly, the maturation of auditory circuits is influenced by “spontaneous,” i.e. sensory-independent, neuronal activity. Action potentials that are not initiated by input from the external environment have been described in ACx in a period starting shortly after birth and lasting until hearing onset at the opening of the auditory canal (Wang and Bergles 2015), when stimuli-evoked responses initiate shaping the organization of nascent circuits with consequent increase of cell desynchronization (Wang and Bergles 2015; Meng et al. 2020; Meng et al. 2021). Using macroscopic calcium imaging on both the inferior colliculus and auditory cortex before hearing onset, it has been shown that neurons responsible for processing similar frequencies of sound exhibit highly synchronized activity at this early developmental age (Babola et al. 2018), providing a mechanism for the activity-dependent refinement and stabilization of synaptic connections within specific frequency ranges.

Despite the prominent role of USVs in mouse social behaviors and communication, no evidence is available on the neuronal representation of USVs early in life. Previous studies have mainly focused on ACx tonotopy development and neuronal representation of pure tones (<32 kHz). However, conspecific vocalizations differ from pure sounds not only for their complex spectrotemporal structure but also for their natural incentive salience (Sangiamo et al. 2020), leaving several open questions on the ability to detect and ultimately interpret USVs during development.

How and when does the capacity to respond to a USV presentation develop? Are different syllables composing a USV bout differently represented in the developing ACx?

In the present study, we addressed these questions by employing in vivo two-photon Ca2+ imaging in anesthetized mice over four developmental windows and characterized single-cell responses to different sound categories. Specifically, we analyzed layer 2/3 (L2/3) ACx representation of USVs from hearing onset (~P12 in mice) to P21 and compared responses to isolated male syllables, known to impact adult behavior when presented during early development (Hammerschmidt et al. 2009; Musolf et al. 2010; Asaba et al. 2014), with pure tones (ranging from 4 to 90 kHz) and with up- (70 to 90 kHz) and down- (90 to 70 kHz) HF-modulated sweep presentation. Moreover, we examined spontaneous activity and network correlation at different ages and evaluated the relationship between spontaneous and sound-evoked activity.

Our results show that ACx L2/3 neurons are tuned to respond to USV syllables as early as P12 to P13. The fraction of neurons that respond to a single syllable increases during development; however, contrary to pure-tone responses, we did not detect an age-related preference for a specific syllable feature. Finally, through the analysis of pairwise cell correlations of both spontaneous and evoked neuronal activity, we demonstrate that clusters of neurons involved in processing similar simple (e.g. pure tones) or complex (e.g. syllables) sounds display higher correlated spontaneous activity after the opening of the auditory canal, coinciding with the initiation of sensory-evoked network activation.

## Materials and methods

### Animals

Experimental procedures involving animals were approved by the University of Turin Ethical Committee and by the National Council on Animal Care of the Italian Ministry of Health (authorization # 422/2022-PR). All experiments were conducted according to the guidelines of the European Communities Council Directive of November 24, 1986 (86/609/EEC). The animals were housed under a 12-h light: dark cycle in individually ventilated cages. Two-photon imaging experiments were performed on C57BL/6j mice (either males or females) for 12 days after birth (P12) to 21 days (P21). Breeding cages were organized with a single adult male bred with one or two adult females. Newborn pups were kept in the cage until the day of the experiment.

### Viral injection

To express the calcium indicator GCaMP in the neurons of the auditory cortex, the adeno-associated virus (AAV) pGP-AAV-syn-jGCaMP7s-WPRE (AAV1; Addgene) was injected (1:10 in saline) in newborn mice at P0 to P1 as previously reported (Zucca et al. 2017). Briefly, P0 to P1 mice were deeply anesthetized by hypothermia, placed on a custom-made stereotaxic apparatus, and kept at 4 °C throughout the entire surgery. The skull was exposed by a small skin incision over the auditory cortex, and ~250 nl of viral suspension was injected using a glass capillary at stereotaxic coordinates of 1 mm posterior and 1.5 mm lateral to the bregma and 0.1 to 0.2 mm depth to target superficial layers. The capillary was kept in place for 2 min before retraction. The skin was then sutured, and the pup was revitalized under an infrared heating lamp.

### USV recording

USVs were recorded from two adult male C57BL/6j mice following acute exposure to conspecific female odors. To elicit reliable USVs, male mice were first exposed to female mice for at least 3 consecutive days. On the fourth day, male mice were exposed only to female scent marks. Scent marks consisted of a mixture of nesting and bedding materials from a single cage containing at least two adult female mice. Females were housed in clean cages, and scent marks were freshly collected the morning of the following day. To record USVs, male mice were first habituated to the soundproof recording chamber in their cage for 15 min, after which the female stimulus was placed in the cage, and USVs were recorded using a calibrated ultrasonic microphone (UltrasoundGate CM16/CMPA, Avisoft) for a total time of 15 min.

### Acoustic stimuli

**Fig. 1:**
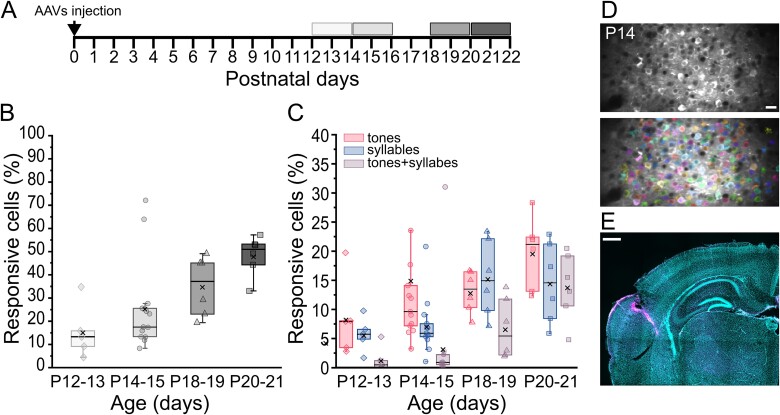
Response properties of L2/3 neurons in the ACx recorded by two-photon Ca2+ imaging. (A) Experimental protocol. P0 mice were injected in the left ACx with AAV1.hsynGCaMP7s, and both spontaneous and evoked activity was recorded in four developmental windows: P12 to P13, P14 to P15, P18 to P19, and P20 to P21. (B, C) Overall fraction of responsive cells (B) and percentage of cells responding to tones, syllables, or both stimuli (C), in the four developmental windows. For each age, the box plots show the median (horizontal black line) and the average value (black cross). The overall fraction of responsive cells increases during development (B; Kruskal–Wallis ANOVA, *P* = 0.00776) as well as the percentage of cells responding to pure tones, syllables, or both (C; Kruskal–Wallis ANOVA, *P* = 0.04641 for tones; *P* = 0.04982 for syllables; *P* = 0.00203 for tones and syllables). P12 to P13 *n* = 5; P14-P15 *n* = 13; P18 to P19 *n* = 6; P20 to P21 *n* = 6. (D) Example of an FOV showing GCaMP7s expression in ACx L2/3 neurons (top panel) and the same FOV with cells detected by Suite2p (Pachitariu et al. 2017) labeled with different colors (bottom panel). Scale bar = 20 μm. E) A posteriori confirmation of the imaged area. Example of a coronal section from a P14 mouse brain, showing rhodamine labeling in the auditory cortical area and counterstained with DAPI (blue). Scale bar = 500 μm.

**Fig. 2:**
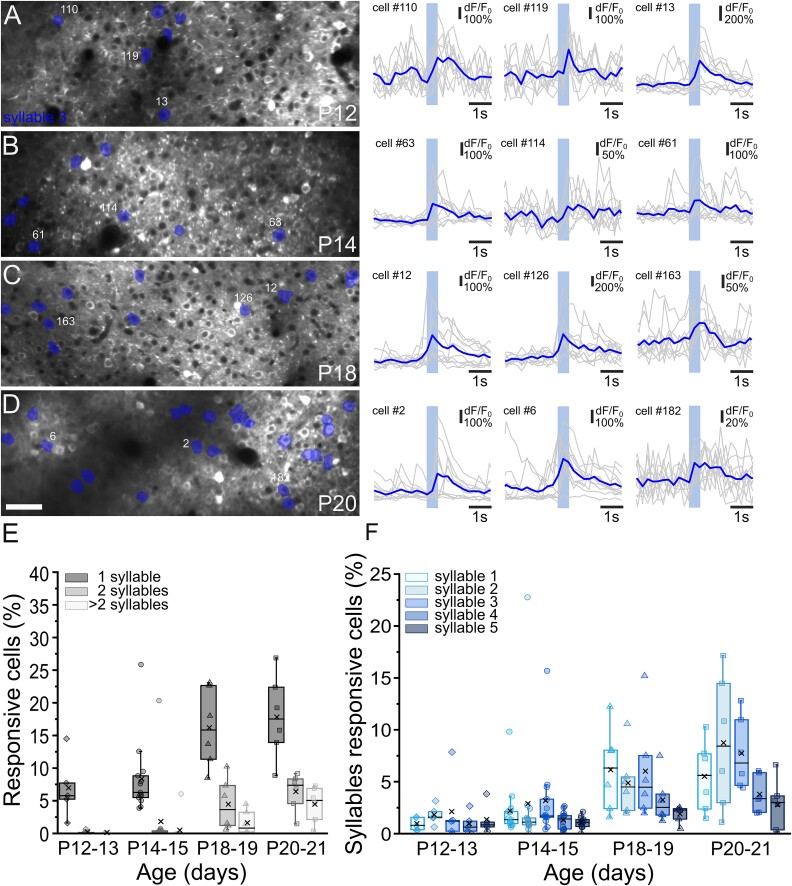
Responses to syllables during development. (A–D) *Left panels*: Examples of FOVs showing GCaMP7s expression in ACx L2/3 neurons at P12 (A), P14 (B), P18 (C), and P20 (D). Cells responding to syllable 3 are color-coded. Scale bar = 50 μm. *Right panels*: Fluorescence signals (dF/*F*_0_) over time from three representative cells responding to playback of syllable 3 at different developmental ages. Gray lines represent a single trial response. Blue lines represent the average response to 10 stimulus presentations (colored rectangle). (E, F) Percentage of cells responding to one syllable, two syllables, or more than two syllables (E) and a fraction of cells preferentially responding to syllables 1 to 5 (F) in the four developmental windows. For each age, the box plots show the median (horizontal black line) and the average value (black cross). No preference toward a syllable was identified (Kruskal–Wallis ANOVA; P12 to P13: *P* = 0.5459, *n* = 5; P14 to P15: *P* = 0.152, *n* = 13: P18 to P19: *P* = 0.1105, *n* = 6; P20 to P21: *P* = 0.09984, *n* = 6).

**Fig. 3:**
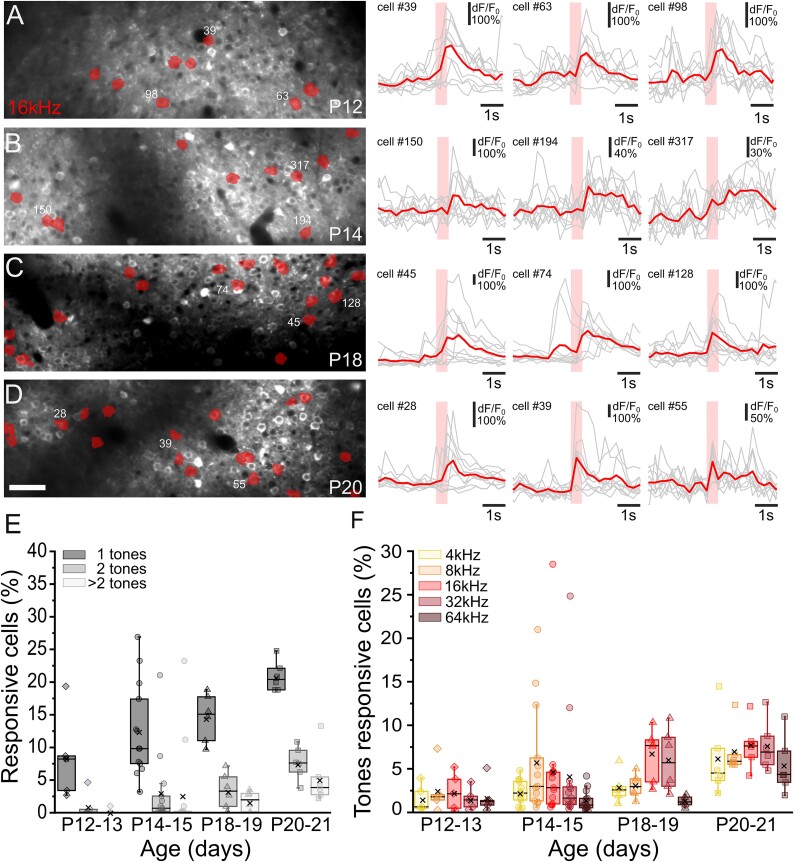
Responses to pure tones during development. (A–D) *Left panels*: Examples of FOVs showing GCaMP7s expression in ACx L2/3 neurons at P12 (A), P14 (B), P18 (C), and P20 (D). Cells responding to 16 kHz tonal stimulus are color-coded. Scale bar = 50 μm. *Right panels*: Fluorescence signals (dF/F_0_) over time from three representative cells responding to 16 kHz tonal presentation at different developmental ages. Gray lines represent a single trial response. Red lines represent the averaged response to 10 stimulus presentation (colored rectangle). (E, F) Percentage of cells responding to one tone, two tones, or more than two tones (E) and the fraction of cells preferentially responding to 4, 8, 16, 32, and 64 kHz (F), in the four developmental windows. For each age, the box plots show the median (horizontal black line) and the average value (black cross). No preference toward a tone was identified at P12 to P13 (Kruskal–Wallis ANOVA, *P* = 0.96504, *n* = 5). P14 to P15 and P18 to P19 show preferential responses to 4 to 16 kHz or 16 to 32 kHz respectively (Kruskal–Wallis ANOVA, P12 to P13: *P* = 0.02941, *n* = 13; P18 to P19: *P* = 0.00193, *n* = 6). At P20 to P21, no preference was detected (Kruskal–Wallis ANOVA, *P* = 0.28784).

**Fig. 4:**
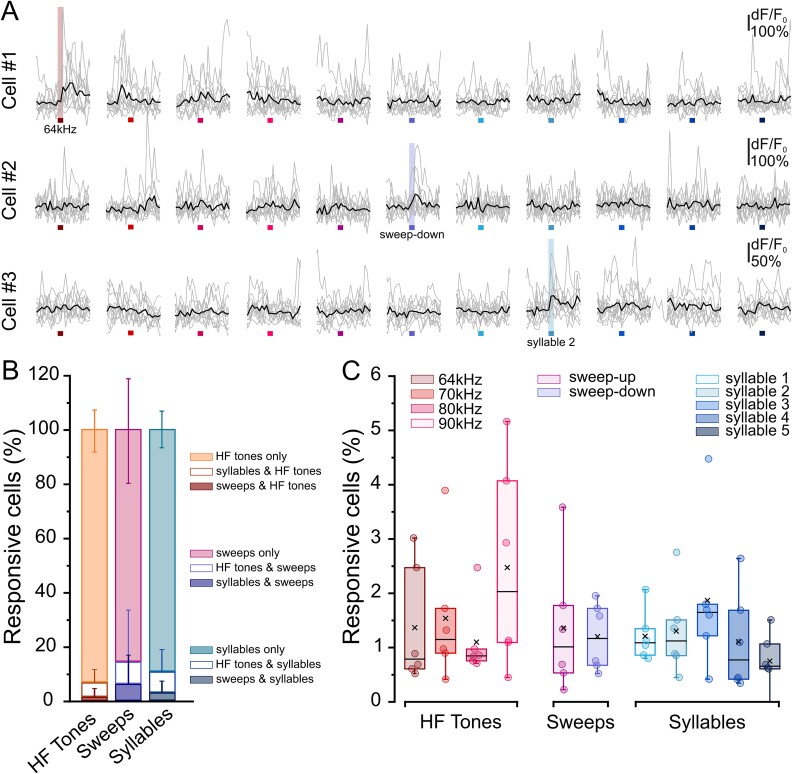
Characterization of ACx L2/3 cells’ responses to an HF sound presentation at P14 to P15. (A) Fluorescence signals (dF/*F*_0_) over time from three representative cells responding to 64 kHz (cell #1), sweep-down (cell #2), and syllable 2 (cell #3). Stimulus presentation lasts 500 ms. Gray lines represent a single-trial response to different HF stimuli (each color represents a stimulus: 64, 70, 80, and 90 kHz, sweep-up, sweep-down, syllable 1, syllable 2, syllable 3, syllable 4, syllable 5). Black lines represent the average response to 10 stimulus presentations. (B) Fraction of cells responding to “HF tones,” “sweeps,” and “syllables” and relative overlapping responses. Values are expressed as average ± sem; *n* = 6. C) Percentage of cells responding to: 64, 70, 80, and 90 kHz, sweep-up, sweep-down, syllable 1, syllable 2, syllable 3, syllable 4, and syllable 5. Box plots show the median (horizontal black line) and the average value (black cross). No difference in the proportion of cells responding to HF pure tones, up- and down-sweeps, and playback of HF syllables was identified (Kruskal–Wallis ANOVA *P* = 0.65171, *n* = 6).

Sound-evoked responses were triggered using a set of custom-made stimuli. Pure tones (Figs. 1 and 3), consisted of a sequence of five 500-ms-long sound stimuli at a single frequency ranging from 4 to 64 kHz and spaced one octave each (4, 8, 16, 32, and 64 kHz), with a 10-ms fade-in and fade-out window. The five different pure tones were randomly presented 10 times each, with an interstimulus interval of 5 s. Syllable stimuli (Figs. 1 and 2) were generated by selecting five different syllables from the USV recordings of two male C57BL/6j adult mice. The full USV recorded signal was first filtered with a high-pass filter at 40 kHz, and noise reduction was applied to the filter trace (threshold: −70 dB, reduction: −140 dB). Syllables were cut from cleaned USV traces for a total length of 30 ms each. Each stimulus consisted of five repetitions of the same syllable spaced 70 ms each, resulting in a total stimulus length of 500 ms. All five syllables were presented 10 times in a random sequence with an interstimulus interval of 5 s. HF pure tones (Fig. 4) were generated by concatenating five 30-ms-long pure sounds with a 2-ms fade-in and fade-out window and spaced 70 ms each for a total stimulus length of 500 ms. Three different frequencies were chosen (70, 80, and 90 kHz) to cover the frequency range of USVs. In addition, up and down sweeps were similarly generated, ranging from 70 to 90 kHz and 90 to 70 kHz, respectively. Each sweep lasted 30 ms with a 2-ms fade-in/out and was repeated five times with 70-ms intervals, for a total stimulus length of 500 ms. Both HF and sweep stimuli were presented 10 times in a random sequence with an interstimulus interval of 5 s. Audio files (.wav) were generated using a custom-made MatLab script.

### Two-photon calcium imaging

To collect sound-evoked responses in layer 2/3 neurons of the mouse auditory cortex, two-photon calcium imaging was performed on young (P12 to P21) mice injected with the AAVs expressing the calcium indicator GCaMP7s at P0 to P1. Mice were first deeply anesthetized with a mixture of Ketamine/Xylazine (50 mg/kg, 4 mg/kg intraperitoneal administration). The skin was then removed to expose the skull, and a 3D-printed head plate was attached to the skull with surgical glue (VetBond) and dental cement, centered over the coordinates of the ACx (1 to 2 mm AP from Bregma, 3 to 4 mm ML from midline, depending on age). A small craniotomy (~300 × 300 μm) was opened on top of the ACx, carefully maintaining the dura intact. The surface of the brain was kept moist with normal N-2-hydroxyethylpiperazine-N′-2-ethanesulfonic acid (HEPES)-buffered artificial cerebrospinal fluid, and body temperature was maintained at 37 °C with a heating pad. The depth of anesthesia was monitored by checking the respiration rate and reactions to pinching the tail and toe. To collect calcium signals, animals were placed under a standard laser scanning two-photon microscope (Nikon A1RMP) coupled to a Chameleon Ultra II (Coherent Santa Clara, CA, λexc = 920 nm). The activity of layer 2/3 neurons was monitored by imaging GCaMP fluorescence via a 16× objective (Nikon CFI75 LWD 16xW NIR A.N.0,80 d. l. 3,0 mm). Signals were acquired by collecting temporal series (t-series) images at an acquisition frame rate of 4 Hz and laser power ranging between 20 and 50 mW. Sound responses were evoked by placing an ultrasonic speaker (Ultrasonic Speaker Vifa, Avisoft) connected to an ultrasonic playback interface (UltraSoundGate Player 116H) controlled by a digital trigger from the two-photon system. The speaker was placed approximately 10 cm from the contralateral right ear of the mouse, and neuronal activity was collected from the left ACx. Stimulus intensity was calibrated by placing the ultrasonic microphone in the same position as the animal under a two-photon microscope.

### Rhodamine labeling of the recorded region for ACx anatomical confirmation

The fluorescent dye rhodamine was used to check for correct anatomical localization of two-photon recordings in ACx. At the end of each experiment, a glass capillary was stained with rhodamine and gently inserted into the imaged craniotomy. Each mouse was then sacrificed, and the brains were extracted and left in a 4% paraformaldehyde in Phosphate-buffered saline (PBS, pH 7.4) overnight for tissue fixation. Fixed brains were socked in a 30% sucrose PBS solution for cryoprotection, and coronal slices (50 μm thick) were later cut and sequentially collected. Slices were stained with 4′,6-diamidino-2-phenylindole (DAPI, 1:1,000, 20 min at RT), mounted on a glass slide with Mowiol mounting medium, and images were acquired via a standard confocal microscope. ACx anatomical confirmation was assessed by checking the rhodamine fluorescent signal along the capillary track in slices aligned with the reference mouse brain atlas (Kronman et al. 2023; https://kimlab.io/brain-map/epDevAtlas/) for ACx localization.

### Two-photon analysis: sound-evoked responses

To assess sound-evoked responses, fluorescent signals from layer 2/3 neurons imaged via two-photon microscopy were extracted using the Python-based software Suite2P (Pachitariu et al. 2017). T-series images from the same field of view (FOV) were concatenated, and regions of interest (ROIs) corresponding to single neurons were automatically identified and manually checked. For each identified ROI, the fluorescent signal and neuropil signal were extracted and stored for subsequent analysis using a custom-made MatLab script. Each signal was first adjusted for neuropil contamination by subtracting 70% of the neuropil signal from the raw cell fluorescence signal (Chen et al. 2013), and the dFF was calculated as follows:


$$ dFF=\frac{F-F0}{F0} $$

where *F* is the raw fluorescent signal corrected for neuropil contamination and *F*0 is the median value of the raw fluorescence calculated across the entire recording.

To characterize sound-evoked responses, calcium traces were aligned to the stimulus presentation, and a time window of 5 s before and after stimulus onset was considered. Neuronal responsiveness was assessed following a previously applied method (Schiavo et al. 2020). Briefly, for each neuron, all 10 repetitions of the same stimulus were considered, and the area under the fluorescence curve was calculated for each trial considering 1 s after stimulus onset (POST) with a 250-ms blank window and 1 s before stimulus onset (PRE). A neuron was classified as responsive if there was a significant difference by comparing the PRE and POST values of the fluorescence area using the parametric paired Student’s *t*-test (two tails) and if the average response calculated in the POST window was higher than the 1.5*STD calculated in the PRE window. The fraction of responsive neurons was calculated for each single animal as responsive neurons across all FOVs divided by the total number of cells recorded from the same animal. To evaluate the best frequency response, only cells responsive to at least one pure tone were considered, and the stimulus with the highest average dFF peak response was considered as the best frequency.

### Two-photon analysis: spontaneous activity

To evaluate the spontaneous activity of layer 2/3 neurons and the relationship between spontaneous and sensory-evoked activity, the basal neuronal activity for each neuron was collected in a time window of 1 min before the start of acoustic stimulation. The raw fluorescence was extracted for each single cell using the method described above. To evaluate the correlation coefficient across paired cells, the Pearson correlation coefficient was calculated considering the whole basal neuronal activity across all neurons recorded within the same FOV. The distribution of all correlation coefficients was calculated for each single animal and averaged across animals of the same age group. To evaluate how the correlation coefficient changes as a function of neuronal distance, we calculated the distance between paired neurons of the same FOV by considering the centroid coordinates obtained from the ROIs identified by Suite2P. Cell distance was then binned (bin size: 10 μm), and the average correlation coefficient was calculated by averaging the correlation coefficients of cells from the same animal with a distance within the distance bin range. Distributions were calculated and averaged across FOVs from animals of the same age range. To assess the average correlation across responsive and nonresponsive cells, cells of the same FOV sharing the response for the same stimuli were pooled together, and their correlation coefficient for spontaneous activity was calculated. Quantification was performed considering each cell across all FOVs and all animals.

### Statistics

The exclusion/inclusion criteria were based only on technical and anatomical issues. Specifically, recordings with technical issues (e.g. movement during calcium imaging recordings) or in which a posteriori analysis of rhodamine staining resulted to be noncentered on the auditory cortex were excluded from the analysis. All recordings with no technical issues and anatomically centered on the auditory cortex were included.

**Fig. 5:**
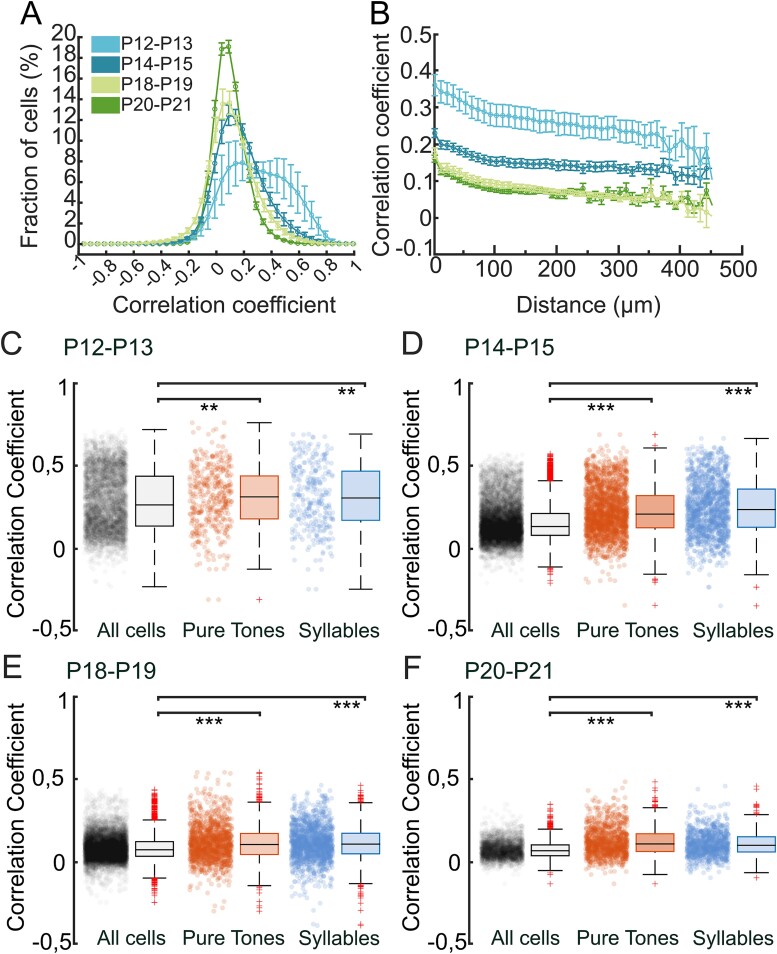
Sound-evoked responses align to correlated spontaneous activity. (A, B) Distribution of L2/3 neurons pairwise correlation in ACx (A, bin: 0.05), and average pairwise correlations as a function of cells distance (B, bin: 10 μm) at P12 to P13, P14 to P15, P18 to P19, and P20 to P21. Cell correlation decreases with age and depends on their distance. Values are expressed as average ± sem. P12 to P13 *n* = 5; P13 to P15 *n* = 13; P18 to P19 *n* = 6; P20 to P21 *n* = 6. (C–F) Distribution of pairwise correlation among L2/3 cells, and among cells responsive to pure tones and syllables at different developmental ages. Box plots show the median (horizontal black line). Red crosses represent the outliers. ^*^^*^P < 0.01, ^*^^*^^*^P < 0.001, Wilcoxon rank sum test. P12 to P13: *n* = 4,694 (all cells), *n* = 451 (pure tones), *n* = 326 (syllables). P14 to P15: *n* = 10,938 (all cells), *n* = 2,149 (pure tones), *n* = 1,390 (syllables). P18 to P19: *n* = 7,652 (all cells), *n* = 1,398 (pure tones), *n* = 1,520 (syllables). P20 to P21: *n* = 3,370 (all cells), *n* = 1,107 (pure tones), *n* = 958 (syllables).

Graphs are represented using both the median and/or the average values and showing maximum, minimum, and outlier values, with the exception of Figs. 4B and 5A and B, where values are expressed as average ± sem. Nonparametric Kruskal–Wallis Analysis of Variance (ANOVA) and Wilcoxon signed-rank tests were used when comparing median values. Statistical analysis was performed using MatLab or OriginLab software.

## Results

### Layer 2/3 neurons in the auditory cortical area respond to HF complex syllables at hearing onset

To investigate the response of L2/3 neurons to USVs during development, we performed in vivo two-photon functional imaging in the auditory cortex of P12 to 21 C57BL/6j mice. We exposed them to a subset of syllables isolated from male mouse USVs and pure tones and quantified the number of responsive cells as well as the proportion of neurons preferentially responding to syllables, pure tones, and both stimuli (Fig. 1). We focused on four developmental windows (Fig. 1A–C), starting from the time window that follows the opening of the auditory canal (Anthwal and Thompson 2016; Ehret 1983; Mikaelian and Ruben 1965) up to P21, corresponding to the functionally identified late critical period for audition (Jouhaneau and Bagady 1984; Zhang et al. 2001; Yang et al. 2012; Meng et al. 2020).

P0 mice were injected in the left ACx (Calhoun et al. 2023) with AAVs driving the GCaMP7s calcium indicator under the hSynapsin promoter (Dana et al. 2019; Fig. 1D), and both their spontaneous activity and sound-evoked responses were recorded. Sound stimuli consisted of the playback of syllables isolated from conspecific USVs (syllable 1: short; syllable 2: step down; syllable 3: up-frequency modulation; syllable 4: flat; syllable 5: step down; frequency range: 75 to 100 kHz; Fonseca et al. 2021). For syllable presentation, we used a sound level between 55 and 65 dB, which we show was the intensity of male emitted USVs (Supplementary Fig. 1). On the same FOV, the response to pure tones presentation was also recorded (4, 8, 16, 32, and 64 kHz, sound intensity = 70 to 80 dB; Romero et al. 2020). At the end of the recording phase, rhodamine was injected into the central portion of the craniotomy, and all brains were withdrawn. For each brain, we confirmed that the recordings were performed in the target area by analyzing a posteriori rhodamine expression (Fig. 1E, see Materials and methods for details).

We first defined responsive cells as any cell showing both (i) an average response to 10 presentations of the same stimulus above a threshold set at 1.5 SD of the baseline averaged activity and (ii) a significant increase in the area under the fluorescent curve after stimulus onset (Schiavo et al. 2020; see Materials and methods). On the total number of detected cells (Fig. 1D, bottom panel), we found that the percentage of cells responding to an auditory stimulus (either syllables and/or tones) increased during development (Kruskal–Wallis ANOVA, *P* = 0.00776) from 13% at P12 to P13 (median; *n* = 5 animals) to 17% at P14 to P15 (median; *n* = 13 animals), 37% at P18 to P19 (median; *n* = 6 animals), and 51% at P20 to P21 (median; *n* = 6 animals; Fig. 1B), consistent with previous calcium imaging and microelectrode recordings (Zhang et al. 2001; Meng et al. 2020). We next evaluated the proportion of responsive cells preferentially activated by syllables or pure tones and the proportion of cells responding to both types of stimuli in different developmental windows. The percentage of cells responding only to syllables or tones increased during development from 6% and 8% at P12 to P13 (median, *n* = 5 animals) to 15% and 21% at P20 to P21 (median, *n* = 6 animals) for syllables and tones, respectively (Kruskal–Wallis ANOVA, *P* = 0.04641 for tones; *P* = 0.04982 for syllables; Fig. 1C). Similarly, later developmental ages (P18 to P19 and P20 to P21) also exhibited an increasing fraction of cells responding to both types of stimuli that set at 14% at P20 to P21 (Kruskal–Wallis ANOVA, *P* = 0.00203). Interestingly, while responses to tones constantly increased from P12 to P21, syllable-evoked responses largely raised at P18 to P21, consistent with previous data in rats, showing that ultrasound frequency representation is slightly delayed compared with pure-tone low-frequency responses (Kim and Bao 2013).

### A subpopulation of L2/3 cells in the auditory area preferentially responds to complex sound features

Having observed syllable-evoked activation in the auditory cortex as early as P12 to P13, we next characterized the response of single neurons in ACx L2/3 to the playback of different types of syllables during development (Fig. 2). Single syllables were isolated from USVs emitted by adult C57BL6/j male mice when exposed to female urine and played-back at 55 to 65 dB using a speaker positioned 10 cm from the contralateral mouse ear (see Materials and methods for details in recording and playback procedures). Syllables were chosen on the basis of their differences in average frequency, shape, and length (Supplementary Fig. 1). To assess whether L2/3 cells in ACx are tuned to syllable identity, we first quantified the percentage of neurons responsive to one, two, or more syllables. At all developmental stages, we found that most of the neurons responded to one syllable only (Fig. 2A–E). In particular, at P12 to P13 and P14 to P15, almost all L2/3 cells responded to a single syllable, whereas, at later developmental stages, the fraction of cells responsive to two or more stimuli increased (Fig. 2E). We then asked whether L2/3 cells in ACx preferentially respond to a specific syllable type at different developmental stages. We found that the number of responsive cells for each syllable type increased from P12 to P21, but we did not observe a clear preference for any of the syllable types at all ages (P12 to P13: Kruskal–Wallis ANOVA, *P* = 0.5459, *n* = 5; P14 to P15: Kruskal–Wallis ANOVA *P* = 0.152, *n* = 13; P18 to P19: Kruskal–Wallis ANOVA *P* = 0.1105, *n* = 6; P20 to P21: Kruskal–Wallis ANOVA *P* = 0.09984, *n* = 6; Fig. 2F).

To investigate if L2/3 selectivity was restricted to syllables or was a common feature of sound responses, we characterized the responses of all recorded cells to each of the five pure tonal stimuli, ranging from 4 to 64 kHz and separated by one octave each (Fig. 3). Consistent with what was found for syllable presentation, L2/3 cells were mostly selective for one stimulus only, with an increased number of cells responsive to two or more stimuli at later developmental stages (Fig. 3A–E). When looking at the preference for a single tone, at P1 to P13, we did not detect a prevalent response to a specific tone (Kruskal–Wallis ANOVA, *P* = 0.96504, *n* = 5; Fig. 3F). In contrast, in P14 to P15 old mice, L2/3 cells showed preferred responses to low-frequency tonal stimuli between 4 and 16 kHz (Kruskal–Wallis ANOVA *P* = 0.02941, *n* = 13; Fig. 3F), consistent with previous studies showing rapid changes in simple tone responses between hearing onset and P14 to P16 (de Villers-Sidani et al. 2007; Carrasco et al. 2013; Polley et al. 2013; Meng et al. 2020; Chen et al. 2021). At P18 to P19, recorded cells showed a shift in tonal preference toward higher frequencies, between 16 and 32 kHz (Kruskal–Wallis ANOVA *P* = 0.00193, *n* = 6; Fig. 3F), whereas no differences were detected at P20 to P21 (Kruskal–Wallis ANOVA *P* = 0.28784, *n* = 6; Fig. 3F).

P14 is a critical age for tonotopic map formation. Thus, we concentrated on this temporal window to better characterize cells’ responses to HF stimuli. At P14 to P15, we detected approximately 6% of cells responding to syllables (median, Fig. 2E) whose high frequencies reside between 60 and 90 kHz (Supplementary Fig. 1), whereas only 0.8% of cells responded to 64 kHz pure tone stimulus (median, Fig. 3F). To better characterize P14 to P15 responses to HF presentation, we recorded stimulus-evoked activity in response to a set of HF stimuli that resembled syllable characteristics such as frequency range and modulation in time. We exposed P14 to P15 mice to four different HF pure tones, namely, 64, 70, 80, and 90 kHz, as well as to increasing and decreasing HF modulated sweeps (70 to 90 kHz, up-sweep; 90 to 70 kHz, down sweep). The analysis of cell coactivation evoked by HF pure tones, up and down sweeps, and syllables showed low overlap between different stimuli (Fig. 4A and B). Moreover, the proportion of cells responding to different HF pure tones, up and down sweeps, and playback of HF syllables was constant (Kruskal–Wallis ANOVA *P* = 0.65171, *n* = 6; Fig. 4A and C), indicating that at P14 to P15, a subpopulation of ACx L2/3 cells preferentially responded to more complex features such as frequency modulation, which characterize ultrasonic vocal communication.

### L2/3 cells tuned for the same stimulus displayed highly correlated spontaneous activity

Before hearing onset, developing neural circuits spontaneously generate highly correlated activity in distinct spatial and temporal patterns (Wang and Bergles 2015; Babola et al. 2018; Meng et al. 2021). These highly stereotyped bursts of action potentials are fundamental for the correct establishment of adult neural circuits that will be later refined through activity-dependent processes upon ear canal opening (Clause et al. 2014; Müller et al. 2019; Kersbergen et al. 2022). At hearing onset, sensory-evoked network activation induces increased desynchronization of spontaneous activity and concomitant intensification of sensory-evoked correlation (Meng et al. 2020). To assess how spontaneous (i.e. stimulus-independent) synchronized activity changes during development, we calculated the pairwise correlation between cell pairs in L2/3 of the auditory cortex at P12 to P13, P14 to P15; P18 to P19, and P20 to P21 and evaluated the distribution of correlation coefficients in each developmental window (bin: 0.05; Fig. 5A). Moreover, because pairwise correlated activity can depend on the distance between neurons (Levy and Reyes 2012; Ko et al. 2013; Winkowski and Kanold 2013; Liu et al. 2019; Meng et al. 2020), we plotted the average correlations between L2/3 cells as a function of their distance for various developmental windows (bin: 10 μm; Fig. 5B). We found that, as expected, the average pairwise correlation decreased as development proceeded in accordance with the occurrence of desynchronized network activity (Fig. 5A and Supplementary Fig. 2). This increased desynchronization was much more evident for cells located within 150 μm, consistent with previously reported data (Levy and Reyes 2012; Winkowski and Kanold 2013; Meng et al. 2020).

Finally, we asked whether cells responding to the same auditory stimulus also displayed a higher spontaneous activity correlation (Fig. 5C–F). Indeed, it was previously demonstrated that, before hearing onset, groups of neurons aligned to the future tonotopic axis that will process similar frequencies of sounds are highly synchronized through the auditory pathway (Babola et al. 2018). To evaluate the relationship between the spontaneous and sound-evoked activity of cells, we looked at the average correlation of spontaneous activity between cell pairs responding to the same stimulus, either pure tones or syllables, and compared it with that obtained from the total cell pairs (including those responding to the same stimulus). At all ages analyzed, the average spontaneous correlation coefficient from cells responding to the same pure tone or syllable resulted to be higher compared to the control group (P12 to P13: *P* = 0.0050084 Wilcoxon rank sum test, “all cells” vs “pure tones”; *P* = 0.0087954, Wilcoxon rank sum test, all cells” vs “syllables”; *n* = 4694, *n* = 451, *n* = 326, for “all cells,” “pure tones,” and “syllables” respectively; P14-P15: *P* = 4.891e-89, Wilcoxon rank sum test, “all cells” vs “pure tones”; *P* = 4.8114e-71, Wilcoxon rank sum test, “all cells” vs “syllables”; *n* = 10,938, *n* = 2149, *n* = 1390, for “all cells,” “pure tones,” and “syllables,” respectively). Interestingly, this difference was maintained at later developmental windows (P18 to P19: *P* = 1.3129e-44, Wilcoxon rank sum test, “all cells” vs “pure tones”; *P* = 1.6082e-28, Wilcoxon rank sum test, “all cells” vs “syllables”; *n* = 7652, *n* = 1398, *n* = 1520, for “all cells,” “pure tones,” and “syllables,” respectively. P20 to P21: *P* = 4.8953e-20, Wilcoxon rank sum test, “all cells” vs “pure tones”; *P* = 5.0015e-33, Wilcoxon rank sum test, “all cells” vs “syllables” *n* = 3370, *n* = 1,107, *n* = 958, for “all cells,” “pure tones,” and “syllables,” respectively), suggesting that groups of neurons processing similar frequencies of sound, that have been shown to exhibit robust correlated activity prior to hearing onset (Babola et al. 2018), maintain this feature also after the opening of the auditory canal when sensory-evoked network activation initiates.

## Discussion

Many vertebrate species emit sound sequences whose structural complexity communicates information beneficial to the receiver (Wang et al. 1995; Gaucher et al. 2013; So et al. 2020; Lenschow et al. 2022). Mice are no exception and produce HF USVs relevant to social, sexual, and emotional interactions with conspecifics (Holy and Guo 2005; Musolf and Penn 2012). Mouse USVs are auditory signals in the HF range (spanning from 35 to 110 kHz) composed of units of sound called syllables, which differ in their frequency range, structure, and duration (Fonseca et al. 2021). Syllables are arranged in nonrandom sequences often organized in phrases that have been shown to be predictable of specific behaviors (Grimsley et al. 2011; Agarwalla et al. 2020; Agarwalla et al. 2023). USVs are emitted by pups to elicit maternal retrieval (Ehret and Haack 1984; Tasaka et al. 2018) and by adults to facilitate social interaction, for example, during courtship behaviors and mate choice (White et al. 1998; Musolf et al. 2010; Yang et al. 2013; Musolf et al. 2015; Lenschow et al. 2022; Agarwalla et al. 2023). Thus, perceiving and encoding USVs plays a prominent role throughout development, from early life up to adulthood. Interestingly, postnatal exposure to acoustic stimuli, including USVs, can impact behavioral choices in adult subjects. For example, adult female mice show a preference for USVs from nonfamiliar males but only if they were reared with their father (Hammerschmidt et al. 2009; Musolf et al. 2010; Asaba et al. 2014). Nonfamiliar USV preference thus requires a learning phase at an early developmental age, during which exposure to father USVs can impact adult auditory processing, similar to what has been described with pure tones and complex sounds exposure, during specific critical developmental windows (Zhang et al. 2001; Insanally et al. 2009; Insanally et al. 2010; Barkat et al. 2011; Kim and Bao 2013; Meng et al. 2020). Despite the integral role of USV communication in mouse behavior, very little is known about the development of USV hearing capability, and no data are available on when and how USV representation forms during development.

In the present study, we analyzed syllable-evoked activation of layer 2/3 neurons in the auditory cortex of mice in four developmental windows, spanning from hearing onset at the opening of the ear canal (P12 to P13), up to P20 to P21. Responses to syllables were compared with responses to pure tonal stimuli presentation (4 to 90 kHz) and to HF-modulated up and down sweeps. Furthermore, by comparing spontaneous activity with sound-evoked responses, we assessed whether L2/3 cell recruitment to the same stimulus at different developmental stages was influenced by previous correlated spontaneous network oscillations.

We observed that syllable-evoked activity in ACx could be detected at hearing onset (P12 to P13). The fraction of responding cells to syllables increased during development with a sharp increment at P18 to P21. At this age, we also detected a higher fraction of syllable-responding neurons recruited by two or more sound stimuli (either 2 syllables or syllable and pure tone), which were rarely seen at earlier stages but have been previously reported in adult ACx (Carruthers et al. 2013; Kim and Bao 2013). Thus, compared with pure tone responses, which show a constant increase in the fraction of responsive cells from P12 to P21 and that were described to attain adult-like structure already at P14 to P16 in mice (Carrasco et al. 2013; Polley et al. 2013; Chen et al. 2021), USV representation during postnatal development might be slightly delayed, reaching adult-like representation later compared with simple tonal stimuli, consistent with data on the development of complex sound responses, which have been shown to emerge within a month-long window (Insanally et al. 2009; Insanally et al. 2010; Carrasco et al. 2013; Kim and Bao 2013).

The delayed representation of USV and complex sounds overlaps with the time course of early adolescence, a period characterized by increased social play, and with the development of proper cognitive strategies that lead to effective coping with adult situations (Burke et al. 2017; Piekarski et al. 2017; Sachser et al. 2020). In rats, play begins at around 17 days of age and fully mature 10 days later (Palagi et al. 2016; Pellis et al. 2022; Cooper et al. 2023), similar to the development of USV responses (Kim and Bao 2013). Recent studies demonstrated that USVs play a crucial role in communication during play, aiding in role reversals and preventing the escalation of aggression (Kisko et al. 2015; Burke et al. 2020). Specifically, the emission of appetitive 50 kHz USVs is a key communication strategy for maintaining and enhancing playful motivation. This is evidenced by a significant reduction in playful behavior observed in pairs of devocalized rats, as well as in intact rats housed with devocalized counterparts (Kisko et al. 2015). Similarly to rats, mice are also engaged in frequent and prolonged bouts of amicable and playful behavior especially during the socialization period, which lasts in mice from the eye opening (around P15) to around P25 (Williams and Scott 1953; Dyer and Southwick 1974; Terranova and Laviola 2005). The observed increase in USV processing throughout development, as indicated by our data, may be a crucial element of social activity, playing a significant role in the development and refinement of species-specific social behavioral repertoires. Conversely, the earlier development of responses to lower tonal frequencies may be associated with the ability to hear audible vocalizations, which have been reported in the context of predatory defense (Ruat et al. 2022). However, there are no available data on whether exposure to complex sonic vocalizations (as opposed to pure low-frequency tones) emitted under stress and fear conditions induces responses at early developmental stages in the ACx.

Previous studies have reported that in both adult rats and mice, neurons in the ACx responding to pure tonal frequencies within the range of USVs are preferentially recruited by the playback of conspecific vocalizations (Carruthers et al. 2013; Kim and Bao 2013; Calhoun et al. 2023). We found that from P12 to P21, the fraction of cells responding to both syllables and tones increased. This analysis, however, was restricted to tonal stimuli largely below the frequency range that characterizes USVs. Thus, we wondered whether syllable-responding cells could be preferentially activated by pure tones in the frequency range of USVs and/or by up- and down-HF modulated sweeps. At P14 to P15, a critical age for tonotopic map formation, we detected low overlap between responses, with most neurons preferentially responding to a single category of stimuli (i.e. HF pure tones or HF sweeps or syllables). In accordance with the delayed maturation of complex sound responses and previous studies showing rapid expansion of best frequency responses from lower to higher frequencies between hearing onset and young adult age (Insanally et al. 2009; Insanally et al. 2010; Carrasco et al. 2013; Kim and Bao 2013; Polley et al. 2013), responses to multiple categories of HF sounds may be delayed and not yet achieved at P14 to P15. Furthermore, at P12 to P13 and P14 to P15, responses to sound presentation were highly variable among animals, coinciding with a dynamic phase for the development of auditory circuits, during which the rapid maturation of cortical responses is strongly susceptible to external manipulation (Zhang et al. 2001; Zhang et al. 2002; de Villers-Sidani et al. 2007; Kim and Bao 2009; Insanally et al. 2010; Barkat et al. 2011; Polley et al. 2013). The variability across animals was much reduced at P18 to P19 and P20 to P21, consistent with advanced circuit maturation and achievement of adult-like anatomical organization of ACx subfields (Chen et al. 2021; Calhoun et al. 2023).

The organization of the adult mouse ACx consists of two primary regions surrounded by higher-order areas, mainly responding to USV presentation (Stiebler et al. 1997; Tsukano et al. 2015; Liu et al. 2019; Romero et al. 2020). Interestingly, recent findings in mice identified the left secondary auditory area to exhibit lateralized functional activation and be primarily involved in HF tones and vocalization responses (Geissler and Ehret 2004; Levy et al. 2019; Calhoun et al. 2023), similar to what has been seen in birds (Schneider and Woolley 2013) and humans (Norman-Haignere et al. 2015) in processing conspecific communication sounds. Accordingly, for our experiments, we focused on the left ACx; however, how lateralization of USV responses develops and to what extent it is influenced by genetic mechanisms or early experience remains an intriguing future goal.

In the context of cortical development and sensory circuit refinement, spontaneous activity has long been recognized to play an important role in shaping the maturation of sensory systems. This highly synchronized stimulus-independent activity controls numerous aspects of sensory system development, from cell integration to circuit refinement (Kirkby et al. 2013; Leighton and Lohmann 2016; Luhmann et al. 2016; Luhmann and Khazipov 2018; Molnár et al. 2020). In the mouse auditory system, spontaneous activity begins prior to hearing onset and continues for almost 2 weeks after birth, when the arrival of auditory signals from the sensory periphery allows neurons to engage in experience-dependent plasticity (Wang and Bergles 2015). Spontaneous activity originates in the developing cochlea and propagates along the auditory pathway up to the ACx, where it aligns with the future tonotopic axis prior to the initiation of sensory-evoked activity (Babola et al. 2018). Suppression or manipulation of spontaneous activity significantly impacts the tonotopic precision of auditory neuron connectivity and their tuning properties, as well as the final anatomical organization of brain regions devoted to sound processing (Clause et al. 2014; Müller et al. 2019; Kersbergen et al. 2022), suggesting that spontaneous activity itself contains information that guides fundamental properties of auditory development. Here, we assessed how network-coordinated activity is preserved after hearing onset and whether the sensory-dependent maturation of tonotopy contributes to maintaining higher correlated spontaneous activity between neurons responding to the same stimulus.

Our results showed that cell correlation decreased with increasing processing of peripheral auditory stimuli, consistent with previous findings in sensory cortices (Leighton and Lohmann 2016; Martini et al. 2021). From hearing onset, the “adult-like” desynchronized activity is attended by a progressive increase in incoming auditory stimuli, which refines neuronal responsive properties and reinforces connections between networks involved in processing specific sound features. Our data demonstrate that clusters of neurons responding to the same stimulus maintain a higher correlated spontaneous activity throughout development, even once circuits reach adult-like anatomical and functional organization. This indicates that, while the overall cortical network becomes less synchronized, neurons specialized for the same stimulus feature maintain higher levels of synchronization, which might be responsible for the stronger synaptic connectivity observed in later developmental stages or in mature cortical regions (Ko et al. 2013; Ko 2014; Rossi et al. 2020).

Overall, our data demonstrate for the first time that neurons in the mouse ACx can process complex USVs immediately after the opening of the hearing canal and that their representation increases as the brain develops, with neurons responding to the same stimulus organized in fine-scale subnetworks maintaining highly correlated spontaneous activity after circuits attained mature organization. These subnetworks may serve as intrinsic building blocks that sustain specific responses to sensory stimuli into adulthood. Indeed, recurrent patterns of spontaneous activity, which resemble those observed under sensory stimulation, have been documented in other mouse sensory regions, such as the visual cortex (MacLean et al. 2005; MacLean et al. 2006; Miller et al. 2014; Carrillo-Reid et al. 2015). The extent to which these networks undergo experience-dependent modifications during development remains to be investigated. In the auditory cortex, early exposure to pure tones in a brief 3-day window, typically reported as postnatal days 11 to 14, leads to an expanded representation of the tone frequency within the tonotopic map (Zhang et al. 2001; de Villers-Sidani et al. 2007; Han et al. 2007; Kim and Bao 2009; Barkat et al. 2011). Sensitive periods for manipulation have also been identified for other auditory features, including binaural hearing, tuning bandwidth, temporal sequences, frequency modulation, and amplitude modulation (Nakahara et al. 2004; Insanally et al. 2009; Polley et al. 2013; Caras and Sanes 2015; Persic et al. 2020), with representations for basic features consolidating before those for more complex ones (Insanally et al. 2009; de Villers-Sidani and Merzenich 2011; Sanes and Woolley 2011). Based on our data indicating a delayed response to USVs compared to pure tones, the sensitive period for USV responsiveness may be shifted, coinciding with the onset of social play and interaction. Exposure to social stimuli, such as USVs emitted by relatives, during this critical window may influence cortical network dynamics, having a significant role in the development and refinement of social behaviors. Indeed, although social communication in mice is primarily mediated by odors, the maturation of salient chemosensory stimuli (e.g. urine pheromones) occurs only postpuberty (Lombardi et al. 1976; Chamero et al. 2007; Oboti et al. 2017), suggesting that the acoustic channel may play a crucial and prominent role in mediating social behavior during the early critical prepubertal stage.

## Supplementary Material

Zucca_et_al_supplementary_information_bhae438

## References

[ref1] Afrashteh N , Jafari Z, Sun J, Kyweriga M, Mohajerani MH. Functional organization of mouse auditory cortex in response to stimulus complexity and brain state. *bioRxiv*. 2022. 10.1101/2022.08.11.503675

[ref2] Agarwalla S , Arroyo NS, Long NE, O'Brien WT, Abel T, Bandyopadhyay S. Male-specific alterations in structure of isolation call sequences of mouse pups with 16p11.2 deletion. Genes Brain Behav. 2020:19:e12681. 10.1111/gbb.12681. 32558237 PMC7116069

[ref3] Agarwalla S , De A, Bandyopadhyay S. Predictive mouse ultrasonic vocalization sequences: uncovering behavioral significance, auditory cortex neuronal preferences, and social-experience-driven plasticity. J Neurosci. 2023:43:6141–6163. 10.1523/JNEUROSCI.2353-22.2023. 37541836 PMC10476644

[ref4] Anthwal N , Thompson H. The development of the mammalian outer and middle ear. J Anat. 2016:228:217–232. 10.1111/joa.12344. 26227955 PMC4718165

[ref5] Arriaga G , Jarvis ED. Mouse vocal communication system: are ultrasonics learned or innate? Brain Lang. 2013:124:96–116. 10.1016/j.bandl.2012.10.002. 23295209 PMC3886250

[ref6] Asaba A , Okabe S, Nagasawa M, Kato M, Koshida N, Osakada T, Mogi K, Kikusui T. Developmental social environment imprints female preference for male song in mice. PLoS One. 2014:9:e87186. 10.1371/journal.pone.0087186. 24505280 PMC3914833

[ref7] Babola TA , Li S, Gribizis A, Lee BJ, Issa JB, Wang HC, Crair MC, Bergles DE. Homeostatic control of spontaneous activity in the developing auditory system. Neuron. 2018:99:511–524.e515. 10.1016/j.neuron.2018.07.004. 30077356 PMC6100752

[ref8] Barkat TR , Polley DB, Hensch TK. A critical period for auditory thalamocortical connectivity. Nat Neurosci. 2011:14:1189–1194. 10.1038/nn.2882. 21804538 PMC3419581

[ref9] Burke AR , McCormick CM, Pellis SM, Lukkes JL. Impact of adolescent social experiences on behavior and neural circuits implicated in mental illnesses. Neurosci Biobehav Rev. 2017:76:280–300. 10.1016/j.neubiorev.2017.01.018. 28111268

[ref10] Burke CJ , Euston DR, Pellis SM. What do you hear what do you say? Ultrasonic calls as signals during play fighting in rats. Int J Play. 2020:9:92–107. 10.1080/21594937.2020.1720126.

[ref11] Calhoun G , Chen CT, Kanold PO. Bilateral widefield calcium imaging reveals circuit asymmetries and lateralized functional activation of the mouse auditory cortex. Proc Natl Acad Sci USA. 2023:120:e2219340120. 10.1073/pnas.2219340120. 37459544 PMC10372568

[ref12] Caras ML , Sanes DH. Sustained perceptual deficits from transient sensory deprivation. J Neurosci. 2015:35:10831–10842. 10.1523/JNEUROSCI.0837-15.2015. 26224865 PMC4518056

[ref13] Carrasco MM , Trujillo M, Razak K. Development of response selectivity in the mouse auditory cortex. Hear Res. 2013:296:107–120. 10.1016/j.heares.2012.11.020. 23261406

[ref14] Carrillo-Reid L , Miller J-E, Hamm JP, Jackson J, Yuste R. Endogenous sequential cortical activity evoked by visual stimuli. J Neurosci. 2015:35:8813–8828. 10.1523/JNEUROSCI.5214-14.2015. 26063915 PMC4461687

[ref15] Carruthers IM , Natan RG, Geffen MN. Encoding of ultrasonic vocalizations in the auditory cortex. J Neurophysiol. 2013:109:1912–1927. 10.1152/jn.00483.2012. 23324323 PMC4073926

[ref16] Chabout J , Sarkar A, Dunson DB, Jarvis ED. Male mice song syntax depends on social contexts and influences female preferences. Front Behav Neurosci. 2015:9:76. 10.3389/fnbeh.2015.00076. 25883559 PMC4383150

[ref17] Chamero P , Marton TF, Logan DW, Flanagan K, Cruz JR, Saghatelian A, et al. Identification of protein pheromones that promote aggressive behaviour. Nature. 2007:450:899–902. 10.1038/nature05997. 18064011

[ref18] Chen TW , Wardill TJ, Sun Y, Pulver SR, Renninger SL, Baohan A, Schreiter ER, Kerr RA, Orger MB, Jayaraman V, et al. Ultrasensitive fluorescent proteins for imaging neuronal activity. Nature. 2013:499:295–300. 10.1038/nature12354. 23868258 PMC3777791

[ref19] Chen F , Takemoto M, Nishimura M, Tomioka R, Song WJ. Postnatal development of subfields in the core region of the mouse auditory cortex. Hear Res. 2021:400:108138. 10.1016/j.heares.2020.108138. 33285368

[ref20] Clause A , Kim G, Sonntag M, Weisz CJ, Vetter DE, Rűbsamen R, Kandler K. The precise temporal pattern of prehearing spontaneous activity is necessary for tonotopic map refinement. Neuron. 2014:82:822–835. 10.1016/j.neuron.2014.04.001. 24853941 PMC4052368

[ref21] Cooper MA , Grizzell JA, Whitten CJ, Burghardt GM. Comparing the ontogeny, neurobiology, and function of social play in hamsters and rats. Neurosci Biobehav Rev. 2023:147:105102. 10.1016/j.neubiorev.2023.105102. 36804399 PMC10023430

[ref22] Dana H , Sun Y, Mohar B, Hulse BK, Kerlin AM, Hasseman JP, Tsegaye G, Tsang A, Wong A, Patel R, et al. High-performance calcium sensors for imaging activity in neuronal populations and microcompartments. Nat Methods. 2019:16:649–657. 10.1038/s41592-019-0435-6. 31209382

[ref91] de Villers-Sidani E , Merzenich MM. Lifelong plasticity in the rat auditory cortex: basic mechanisms and role of sensory experience. Prog Brain Res. 2011:191:119–131. 10.1016/B978-0-444-53752-2.00009-6. 21741548

[ref92] de Villers-Sidani E , Chang EF, Bao S, Merzenich MM. Critical period window for spectral tuning defined in the primary auditory cortex (A1) in the rat. J Neurosci. 2007:27:180–189. 10.1523/JNEUROSCI.3227-06.2007. 17202485 PMC6672294

[ref23] Dyer DP , Southwick CH. A possible sensitive period for juvenile socialization in mice. Behav Biol. 1974:12:551–558. 10.1016/S0091-6773(74)92471-7. 4476211

[ref75] Ehret G . Development of hearing and response behavior to sound stimuli: Behavioral studies. R. Romand, editor. Development of auditory and vestibular systems, Academic Press, New York; 1983, pp. 211–237.

[ref24] Ehret G , Haack B. Motivation and arousal influence sound-induced maternal pup-retrieving behavior in lactating house mice. Z Tierpsychol. 1984:65:25–39. 10.1111/j.1439-0310.1984.tb00370.x.

[ref25] Fonseca AH , Santana GM, Bosque Ortiz GM, Bampi S, Dietrich MO. Analysis of ultrasonic vocalizations from mice using computer vision and machine learning. elife. 2021:10:10. 10.7554/eLife.59161. PMC8057810 33787490

[ref26] Garcia-Lazaro JA , Shepard KN, Miranda JA, Liu RC, Lesica NA. An overrepresentation of high frequencies in the mouse inferior colliculus supports the processing of ultrasonic vocalizations. PLoS One. 2015:10:e0133251. 10.1371/journal.pone.0133251. 26244986 PMC4526676

[ref27] Gaucher Q , Huetz C, Gourévitch B, Laudanski J, Occelli F, Edeline JM. How do auditory cortex neurons represent communication sounds? Hear Res. 2013:305:102–112. 10.1016/j.heares.2013.03.011. 23603138

[ref28] Geissler DB , Ehret G. Auditory perception vs. recognition: representation of complex communication sounds in the mouse auditory cortical fields. Eur J Neurosci. 2004:19:1027–1040. 10.1111/j.1460-9568.2004.03205.x. 15009150

[ref29] Grimsley JM , Monaghan JJ, Wenstrup JJ. Development of social vocalizations in mice. PLoS One. 2011:6:e17460. 10.1371/journal.pone.0017460. 21408007 PMC3052362

[ref30] Hammerschmidt K , Radyushkin K, Ehrenreich H, Fischer J. Female mice respond to male ultrasonic `songs' with approach behaviour. Biol Lett. 2009:5:589–592. 10.1098/rsbl.2009.0317. 19515648 PMC2781958

[ref31] Han YK , Kover H, Insanally MN, Semerdjian JH, Bao S. Early experience impairs perceptual discrimination. Nat Neurosci. 2007:10:1191–1197. 10.1038/nn1941. 17660815

[ref32] Holy TE , Guo Z. Ultrasonic songs of male mice. PLoS Biol. 2005:3:e386. 10.1371/journal.pbio.0030386. 16248680 PMC1275525

[ref33] Insanally MN , Köver H, Kim H, Bao S. Feature-dependent sensitive periods in the development of complex sound representation. J Neurosci. 2009:29:5456–5462. 10.1523/JNEUROSCI.5311-08.2009. 19403813 PMC2717948

[ref34] Insanally MN , Albanna BF, Bao S. Pulsed noise experience disrupts complex sound representations. J Neurophysiol. 2010:103:2611–2617. 10.1152/jn.00872.2009. 20200123 PMC2867562

[ref35] Jouhaneau J , Bagady A. Effect of early auditory stimulation on the choice of acoustical environment by adult Swiss albino mice (Mus musculus). J Comp Psychol. 1984:98:318–326. 10.1037/0735-7036.98.3.318. 6478786

[ref36] Kersbergen CJ , Babola TA, Rock J, Bergles DE. Developmental spontaneous activity promotes formation of sensory domains, frequency tuning and proper gain in central auditory circuits. Cell Rep. 2022:41:111649. 10.1016/j.celrep.2022.111649. 36384119 PMC9730452

[ref37] Kim H , Bao S. Selective increase in representations of sounds repeated at an ethological rate. J Neurosci. 2009:29:5163–5169. 10.1523/JNEUROSCI.0365-09.2009. 19386912 PMC2717947

[ref38] Kim H , Bao S. Experience-dependent overrepresentation of ultrasonic vocalization frequencies in the rat primary auditory cortex. J Neurophysiol. 2013:110:1087–1096. 10.1152/jn.00230.2013. 23741037 PMC3763088

[ref39] Kirkby LA , Sack GS, Firl A, Feller MB. A role for correlated spontaneous activity in the assembly of neural circuits. Neuron. 2013:80:1129–1144. 10.1016/j.neuron.2013.10.030. 24314725 PMC4560201

[ref40] Kisko TM , Wöhr M, Pellis VC, Pellis SM. From play to aggression: high-frequency 50-kHz ultrasonic vocalizations as play and appeasement signals in rats. In: Social behavior from rodents to humans. Springer, Cham; 2015:30:91–108. 10.1007/7854_2015_432. 26728173

[ref41] Ko H . Functional organization of synaptic connections in the neocortex. Science. 2014:31, 346:555. 10.1126/science.1260780. 25359956

[ref42] Ko H , Cossell L, Baragli C, Antolik J, Clopath C, Hofer SB, Mrsic-Flogel TD. The emergence of functional microcircuits in visual cortex. Nature. 2013:496:96–100. 10.1038/nature12015. 23552948 PMC4843961

[ref43] Kronman FA , Liwang JK, Betty R, Vanselow DJ, Wu Y, Tustison NJ, Bhandiwad A, Manjila SB, Minteer JA, Shin D, et al. Developmental mouse brain common coordinate framework. bioRxiv. 2023. 10.1101/2023.09.14.557789. PMC11494176 39433760

[ref44] Leighton AH , Lohmann C. The wiring of developing sensory circuits-from patterned spontaneous activity to synaptic plasticity mechanisms. Front Neural Circuits. 2016:10:71. 10.3389/fncir.2016.00071. 27656131 PMC5011135

[ref45] Lenschow C , Mendes ARP, Lima SQ. Hearing, touching, and multisensory integration during mate choice. Front Neural Circuits. 2022:16:943888. 10.3389/fncir.2022.943888. 36247731 PMC9559228

[ref46] Levy RB , Reyes AD. Spatial profile of excitatory and inhibitory synaptic connectivity in mouse primary auditory cortex. J Neurosci. 2012:32:5609–5619. 10.1523/JNEUROSCI.5158-11.2012. 22514322 PMC3359703

[ref47] Levy RB , Marquarding T, Reid AP, Pun CM, Renier N, Oviedo HV. Circuit asymmetries underlie functional lateralization in the mouse auditory cortex. Nat Commun. 2019:10:2783. 10.1038/s41467-019-10690-3. 31239458 PMC6592910

[ref48] Liu J , Whiteway MR, Sheikhattar A, Butts DA, Babadi B, Kanold PO. Parallel processing of sound dynamics across mouse auditory cortex via spatially patterned thalamic inputs and distinct areal intracortical circuits. Cell Rep. 2019:27:872–885.e877. 10.1016/j.celrep.2019.03.069. 30995483 PMC7238664

[ref49] Lombardi JR , Vandenbergh JG, Whitsett JM. Androgen control of the sexual maturation pheromone in house mouse urine. Biol Reprod. 1976:15:179–186. 10.1095/biolreprod15.2.179. 963148

[ref50] Luhmann HJ , Khazipov R. Neuronal activity patterns in the developing barrel cortex. Neuroscience. 2018:368:256–267. 10.1016/j.neuroscience.2017.05.025. 28528963

[ref51] Luhmann HJ , Sinning A, Yang JW, Reyes-Puerta V, Stüttgen MC, Kirischuk S, Kilb W. Spontaneous neuronal activity in developing neocortical networks: from single cells to large-scale interactions. Front Neural Circuits. 2016:10:40. 10.3389/fncir.2016.00040. 27252626 PMC4877528

[ref52] MacLean JN , Watson BO, Aaron GB, Yuste R. Internal dynamics determine the cortical response to thalamic stimulation. Neuron. 2005:48:811–823. 10.1016/j.neuron.2005.09.035. 16337918

[ref53] MacLean JN , Fenstermaker V, Watson BO, Yuste R. A visual thalamocortical slice. Nat Methods. 2006:3:129–134. 10.1038/nmeth849. 16432523

[ref54] Martini FJ , Guillamón-Vivancos T, Moreno-Juan V, Valdeolmillos M, López-Bendito G. Spontaneous activity in developing thalamic and cortical sensory networks. Neuron. 2021:109:2519–2534. 10.1016/j.neuron.2021.06.026. 34293296 PMC7611560

[ref55] McMullen NT , Goldberger B, Glaser EM. Postnatal development of lamina III/IV nonpyramidal neurons in rabbit auditory cortex: quantitative and spatial analyses of Golgi-impregnated material. J Comp Neurol. 1988:278:139–155. 10.1002/cne.902780109. 2463295

[ref56] Meng X , Solarana K, Bowen Z, Liu J, Nagode DA, Sheikh A, Winkowski DE, Kao JPY, Kanold PO. Transient subgranular hyperconnectivity to L2/3 and enhanced pairwise correlations during the critical period in the mouse auditory cortex. Cereb Cortex. 2020:30:1914–1930. 10.1093/cercor/bhz213. 31667495 PMC7132924

[ref57] Meng X , Mukherjee D, Kao JPY, Kanold PO. Early peripheral activity alters nascent subplate circuits in the auditory cortex. Sci Adv. 2021:7:eabc9155. 10.1126/sciadv.abc9155. PMC7880598 33579707

[ref58] Mikaelian D , Ruben RJ. Development of hearing in the normal cba-J mouse: correlation of physiological observations with behavioral responses and with cochlear anatomy. Acta Otolaryngol. 1965:59:451–461. 10.3109/00016486509124579. 14198707

[ref59] Miller JE , Ayzenshtat I, Carrillo-Reid L, Yuste R. Visual stimuli recruit intrinsically generated cortical ensembles. Proc Natl Acad Sci USA. 2014:111:E4053–E4061. 10.1073/pnas.1406077111. 25201983 PMC4183303

[ref60] Molnár Z , Luhmann HJ, Kanold PO. Transient cortical circuits match spontaneous and sensory-driven activity during development. Science. 2020:370:eabb2153. 10.1126/science.abb2153. PMC8050953 33060328

[ref61] Müller NIC , Sonntag M, Maraslioglu A, Hirtz JJ, Friauf E. Topographic map refinement and synaptic strengthening of a sound localization circuit require spontaneous peripheral activity. J Physiol. 2019:597:5469–5493. 10.1113/JP277757. 31529505

[ref62] Musolf K , Penn DJ. Ultrasonic vocalizations in house mice: A cryptic mode of acoustic communication. In: Macholan M, Baird SJE, Munclinger P, Pialek J, editors. Evolution of the house mouse. Cambridge: Cambridge University Press; 2012. pp. 253–277.

[ref63] Musolf K , Hoffmann F, Penn DJ. Ultrasonic courtship vocalizations in wild house mice, *Mus musculus musculus*. Anim Behav. 2010:757–764.

[ref64] Musolf K , Meindl S, Larsen AL, Kalcounis-Rueppell MC, Penn DJ. Ultrasonic vocalizations of male mice differ among species and females show assortative preferences for male calls. PLoS One. 2015:10:e0134123. 10.1371/journal.pone.0134123. 26309246 PMC4550448

[ref65] Nakahara H , Zhang LI, Merzenich MM. Specialization of primary auditory cortex processing by sound exposure in the critical period. Proc Natl Acad Sci USA. 2004:101:7170–7174. 10.1073/pnas.0401196101. 15118079 PMC406484

[ref66] Norman-Haignere S , Kanwisher NG, McDermott JH. Distinct cortical pathways for music and speech revealed by hypothesis-free voxel decomposition. Neuron. 2015:88:1281–1296. 10.1016/j.neuron.2015.11.035. 26687225 PMC4740977

[ref67] Oboti L , Trova S, Schellino R, Marraudino M, Harris NR, Abiona OM, et al. Activity dependent modulation of granule cell survival in the accessory olfactory bulb at puberty. Front Neuroanat. 2017:11:44. 10.3389/fnana.2017.00044. 28588456 PMC5440572

[ref68] Pachitariu M , Stringer C, Dipoppa M, Schröder S, Rossi LF, Dalgleish H, Carandini M, Harris KD. Suite2p: beyond 10,000 neurons with standard two-photon microscopy. bioRxiv. 2017. 10.1101/061507.

[ref69] Palagi E , Burghardt GM, Smuts B, Cordoni G, et al. Rough-and-tumble play as a window on animal communication. Biol Rev. 2016:91:311–327. 10.1111/brv.12172. 25619897

[ref70] Pellis SM , Pellis VC, Ham JR, Achterberg EJM. The rough-and-tumble play of rats as a natural behavior suitable for studying the social brain. Front Behav Neurosci. 2022:16:3999. 10.3389/fnbeh.2022.1033999. PMC9623181 36330048

[ref71] Persic D , Thomas ME, Pelekanos V, Ryugo DK, Takesian AE, Krumbholz K, Pyott SJ. Regulation of auditory plasticity during critical periods and following hearing loss. Hear Res. 2020:397:107976. 10.1016/j.heares.2020.107976. 32591097 PMC8546402

[ref72] Piekarski DJ , Johnson CM, Boivin JR, Thomas AW, Lin WC, Delevich K, Galarce EM, Wilbrecht L. Does puberty mark a transition in sensitive periods for plasticity in the associative neocortex? Brain Res. 2017:1654:123–144. 10.1016/j.brainres.2016.08.042. 27590721 PMC5283387

[ref73] Polley DB , Thompson JH, Guo W. Brief hearing loss disrupts binaural integration during two early critical periods of auditory cortex development. Nat Commun. 2013:4:2547. 10.1038/ncomms3547. 24077484 PMC4131765

[ref74] Rao RP , Mielke F, Bobrov E, Brecht M. Vocalization-whisking coordination and multisensory integration of social signals in rat auditory cortex. elife. 2014:3:e03185. 10.7554/eLife.03185. 25485525 PMC4270083

[ref76] Romero S , Hight AE, Clayton KK, Resnik J, Williamson RS, Hancock KE, Polley DB. Cellular and widefield imaging of sound frequency Organization in Primary and Higher Order Fields of the mouse auditory cortex. Cereb Cortex. 2020:30:1603–1622. 10.1093/cercor/bhz190. 31667491 PMC7132909

[ref77] Rossi LF , Harris KD, Carandini M. Spatial connectivity matches direction selectivity in visual cortex. Nature. 2020:588:648–652. 10.1038/s41586-020-2894-4. 33177719 PMC7116721

[ref78] Ruat J , Genewsky AJ, Heinz DE, Kaltwasser SF, Canteras NS, Czisch M, et al. Why do mice squeak? Toward a better understanding of defensive vocalization. iScience. 2022:25:104657. 10.1016/j.isci.2022.104657. 35845167 PMC9283514

[ref79] Sachser N , Zimmermann TD, Hennessy MB, Kaiser S. Sensitive phases in the development of rodent social behavior. Curr Opin Behav Sci. 2020:36:63–70. 10.1016/j.cobeha.2020.07.014. 34337112 PMC8318223

[ref80] Sanes DH , Woolley SM. A behavioral framework to guide research on central auditory development and plasticity. Neuron. 2011:72:912–929. 10.1016/j.neuron.2011.12.005. 22196328 PMC3244881

[ref81] Sangiamo DT , Warren MR, Neunuebel JP. Ultrasonic signals associated with different types of social behavior of mice. Nat Neurosci. 2020:23:411–422. 10.1038/s41593-020-0584-z. 32066980 PMC7065962

[ref82] Scattoni ML , Ricceri L, Crawley JN. Unusual repertoire of vocalizations in adult BTBR T+tf/J mice during three types of social encounters. Genes Brain Behav. 2011:10:44–56. 10.1111/j.1601-183X.2010.00623.x. 20618443 PMC2972364

[ref83] Schachtele SJ , Losh J, Dailey ME, Green SH. Spine formation and maturation in the developing rat auditory cortex. J Comp Neurol. 2011:519:3327–3345. 10.1002/cne.22728. 21800311 PMC3905797

[ref84] Schiavo JK , Valtcheva S, Bair-Marshall CJ, Song SC, Martin KA, Froemke RC. Innate and plastic mechanisms for maternal behaviour in auditory cortex. Nature. 2020:587:426–431. 10.1038/s41586-020-2807-6. 33029014 PMC7677212

[ref85] Schneider DM , Woolley SM. Sparse and background-invariant coding of vocalizations in auditory scenes. Neuron. 2013:79:141–152. 10.1016/j.neuron.2013.04.038. 23849201 PMC3713513

[ref86] So NLT , Edwards JA, Woolley SMN. Auditory selectivity for spectral contrast in cortical neurons and behavior. J Neurosci. 2020:40:1015–1027. 10.1523/JNEUROSCI.1200-19.2019. 31826944 PMC6989003

[ref87] Stiebler I , Neulist R, Fichtel I, Ehret G. The auditory cortex of the house mouse: left-right differences, tonotopic organization and quantitative analysis of frequency representation. J Comp Physiol A. 1997:181:559–571. 10.1007/s003590050140. 9449817

[ref88] Tasaka GI , Guenthner CJ, Shalev A, Gilday O, Luo L, Mizrahi A. Genetic tagging of active neurons in auditory cortex reveals maternal plasticity of coding ultrasonic vocalizations. Nat Commun. 2018:9:871. 10.1038/s41467-018-03183-2. 29491360 PMC5830453

[ref89] Terranova ML , Laviola G. Scoring of social interactions and play in mice during adolescence. Curr Protoc Toxicol. 2005:26:10. 10.1002/0471140856.tx1310s26. 23045110

[ref90] Tsukano H , Horie M, Bo T, Uchimura A, Hishida R, Kudoh M, Takahashi K, Takebayashi H, Shibuki K. Delineation of a frequency-organized region isolated from the mouse primary auditory cortex. J Neurophysiol. 2015:113:2900–2920. 10.1152/jn.00932.2014. 25695649 PMC4416634

[ref93] Wang HC , Bergles DE. Spontaneous activity in the developing auditory system. Cell Tissue Res. 2015:361:65–75. 10.1007/s00441-014-2007-5. 25296716 PMC7046314

[ref94] Wang X , Merzenich MM, Beitel R, Schreiner CE. Representation of a species-specific vocalization in the primary auditory cortex of the common marmoset: temporal and spectral characteristics. J Neurophysiol. 1995:74:2685–2706. 10.1152/jn.1995.74.6.2685. 8747224

[ref95] White NR , Prasad M, Barfield RJ, Nyby JG. 40- and 70-kHz vocalizations of mice (Mus musculus) during copulation. Physiol Behav. 1998:63:467–473. 10.1016/S0031-9384(97)00484-8. 9523885

[ref96] Williams E , Scott JP. The development of social behaviour patterns in the mouse, in relation to natural periods. Behaviour. 1953:6:35–64. 10.1163/156853954X00031.

[ref97] Winkowski DE , Kanold PO. Laminar transformation of frequency organization in auditory cortex. J Neurosci. 2013:33:1498–1508. 10.1523/JNEUROSCI.3101-12.2013. 23345224 PMC3783029

[ref98] Yang EJ , Lin EW, Hensch TK. Critical period for acoustic preference in mice. Proc Natl Acad Sci USA. 2012:109:17213–17220. 10.1073/pnas.1200705109. 23045690 PMC3477391

[ref99] Yang M , Loureiro D, Kalikhman D, Crawley JN. Male mice emit distinct ultrasonic vocalizations when the female leaves the social interaction arena. Front Behav Neurosci. 2013:7:159. 10.3389/fnbeh.2013.00159. 24312027 PMC3832782

[ref100] Zhang LI , Bao S, Merzenich MM. Persistent and specific influences of early acoustic environments on primary auditory cortex. Nat Neurosci. 2001:4:1123–1130. 10.1038/nn745. 11687817

[ref101] Zhang LI , Bao S, Merzenich MM. Disruption of primary auditory cortex by synchronous auditory inputs during a critical period. Proc Natl Acad Sci USA. 2002:99:2309–2314. 10.1073/pnas.261707398. 11842227 PMC122361

[ref102] Zucca S , D'Urso G, Pasquale V, Vecchia D, Pica G, Bovetti S, Moretti C, Varani S, Molano-Mazón M, Chiappalone M, et al. An inhibitory gate for state transition in cortex. elife. 2017:6:6. 10.7554/eLife.26177. PMC5444901 28509666

