# Supplementary material for: Developmental encoding of natural sounds in the mouse auditory cortex
Source: Cereb Cortex. 2024 Nov 6;34(11):bhae438. doi: 10.1093/cercor/bhae438 (PMC11538960; doi:10.1093/cercor/bhae438)
Supplement: Zucca_et_al_supplementary_information_bhae438 [file zucca_et_al_supplementary_information_bhae438.pdf]

Supplementary Figure 1

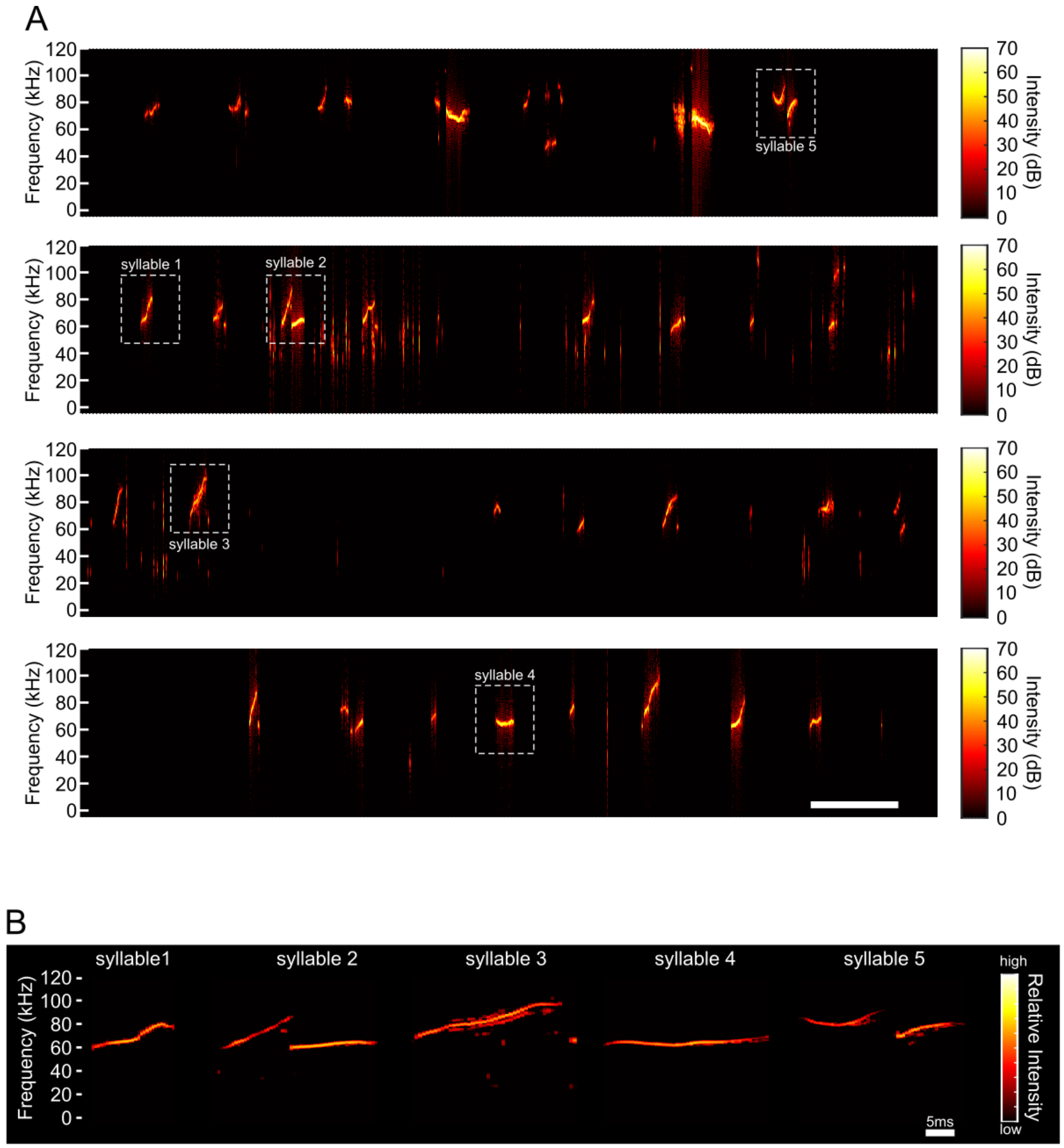

Supplementary Fig. 1. Ultrasonic vocalizations (USVs) emitted by a C57BL6J male mouse. (A) Four examples of USVs emitted by an adult (P60) C57BL6J male mouse when exposed to female urine. USVs were recorded at a sampling frequency of 200 kHz via an ultrasound microphone CM16/CMPA (Avisoft Bioacoustic, Germany), coupled with a recording interface (UltrasoundGate 116Hb, Avisoft Bioacoustic) and recording software (Avisoft Recorder). All recordings were performed in a sound-proof box and USVs were elicited by exposing adult male mice to females' scent marks. (B) Five syllables differing in their length, frequency, and shape were isolated from USV recordings.

Supplementary Figure 2

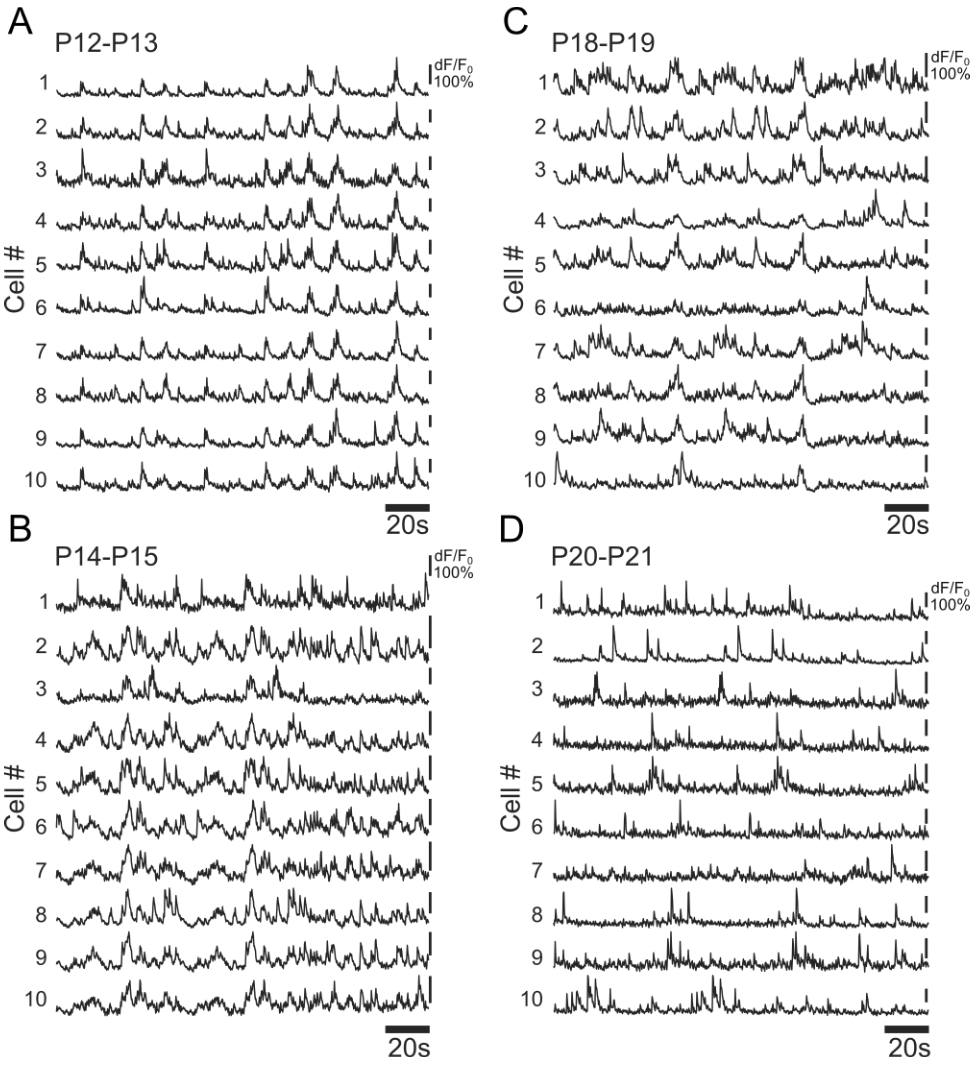

Supplementary Fig. 2. Spontaneous activity in L2/3 neurons desynchronizes while development proceeds. (A-D) Spontaneous fluorescence signals (dF/F_0) over time from 10 representative L2/3 ACx cells, at P12-P13 (A), P14-P15 (B), P18-P19 (C) an
